# A Rare Case of Left Ventricular Noncompaction Discovered During Severe Rhythm Disorder: About a Case Report

**DOI:** 10.1002/ccr3.71673

**Published:** 2025-12-17

**Authors:** Martin Wendlassida Nacanabo, André Arthur Taryètba Seghda, Anna Tall/Thiam, Georges Christian Millogo, Valentin Nobila Yaméogo, André Koudnoaga Samadoulougou, Patrice Zabsonré

**Affiliations:** ^1^ Cardiology Department Bogodogo University Hospital Burkina Faso; ^2^ Cardiology Department Yalgado Ouedraogo University Hospital Burkina Faso

**Keywords:** left ventricle, non‐compaction, tachycardia, trabeculations

## Abstract

The discovery of non‐compaction cardiomyopathy of the left ventricle (NCVG), although rare, is not exceptional. This case involved a 36‐year‐old man admitted with palpitations, exertional dyspnea, hypersudation, and anxiety. The electrocardiogram revealed ventricular tachycardia. Transthoracic Doppler echocardiography and cardiac magnetic resonance imaging revealed hypertrabeculation of the left ventricle, with a ratio of noncompacted to compacted myocardium of 3.04. LVEF was 64%. The diagnosis of ventricular tachycardia revealing noncompaction of the left ventricle complicated by lateral ischemia was accepted. Anti‐arrhythmic treatment with external electric shock enabled sinus rhythm to be achieved with a good reduction in tachycardia.

**Key Message**

This clinical case describes an unusual circumstance in which a rare cardiomyopathy caused by non‐compaction of the left ventricle was discovered. The patient presented with four episodes of ventricular tachycardia resuscitated by electrical cardioversion. He was then put on Amiodarone for maintenance treatment.

## 1 Introduction

Left ventricular noncompaction (LVNC) is a congenital cardiomyopathy characterized by the presence of fetal myocardium with excessive prominence of a network of trabeculations and deep intertrabecular cavities [1]. It is a rare primary cardiomyopathy with an estimated prevalence of 0.014% and a male predominance [2]. Clinical manifestations are highly varied, ranging from asymptomatic forms to deterioration in systolic function, left ventricular dilatation, systemic embolic events, and ventricular arrhythmias or sudden cardiac arrest [3]. Diagnosis of LVNC is based on at least four echocardiographic criteria established by Stöllberger et al. [4] sometimes aided by cardiac MRI. We report a case of LVNC discovered during a severe rhythm disorder in an adult.

## 2 Observation

### 2.1 Case History

A 36‐year‐old chauffeur with a sedentary lifestyle as a cardiovascular risk factor was admitted to the emergency department with palpitations, exertional dyspnea, hypersudation, and anxiety. The patient was transferred to a cardiac intensive care unit.

### 2.2 Examination

Clinical examination revealed arterial hypotension at 90/60 mmHg, tachycardia at 250 beats per minute, polypnoea at 24 cycles per minute, and anxiety. The rest of the physical examination was normal.

### 2.3 Investigation

**FIGURE 1:**
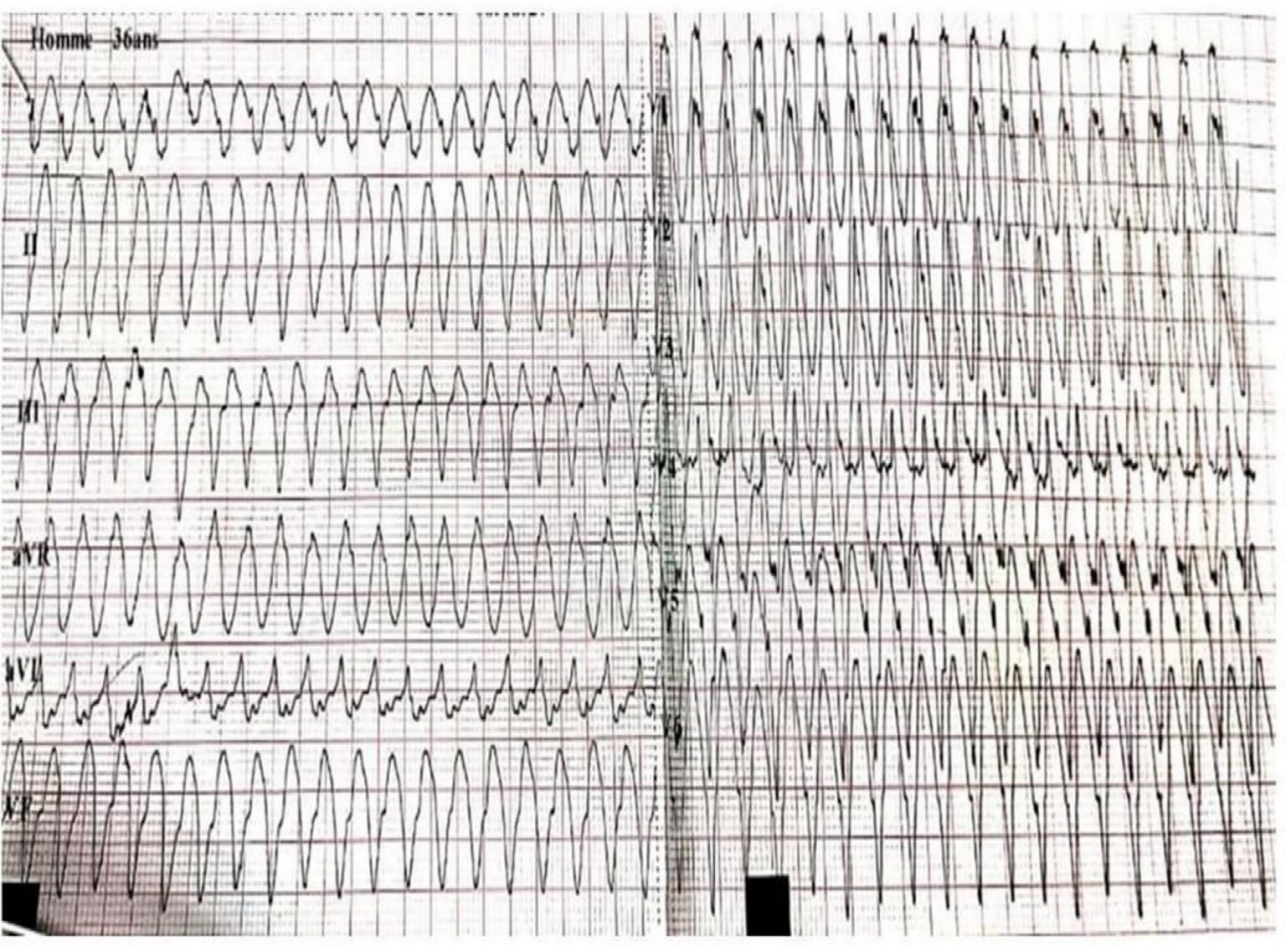
12‐lead electrocardiogram showing wide QRS tachycardia suggestive of ventricular tachycardia.

**FIGURE 2:**
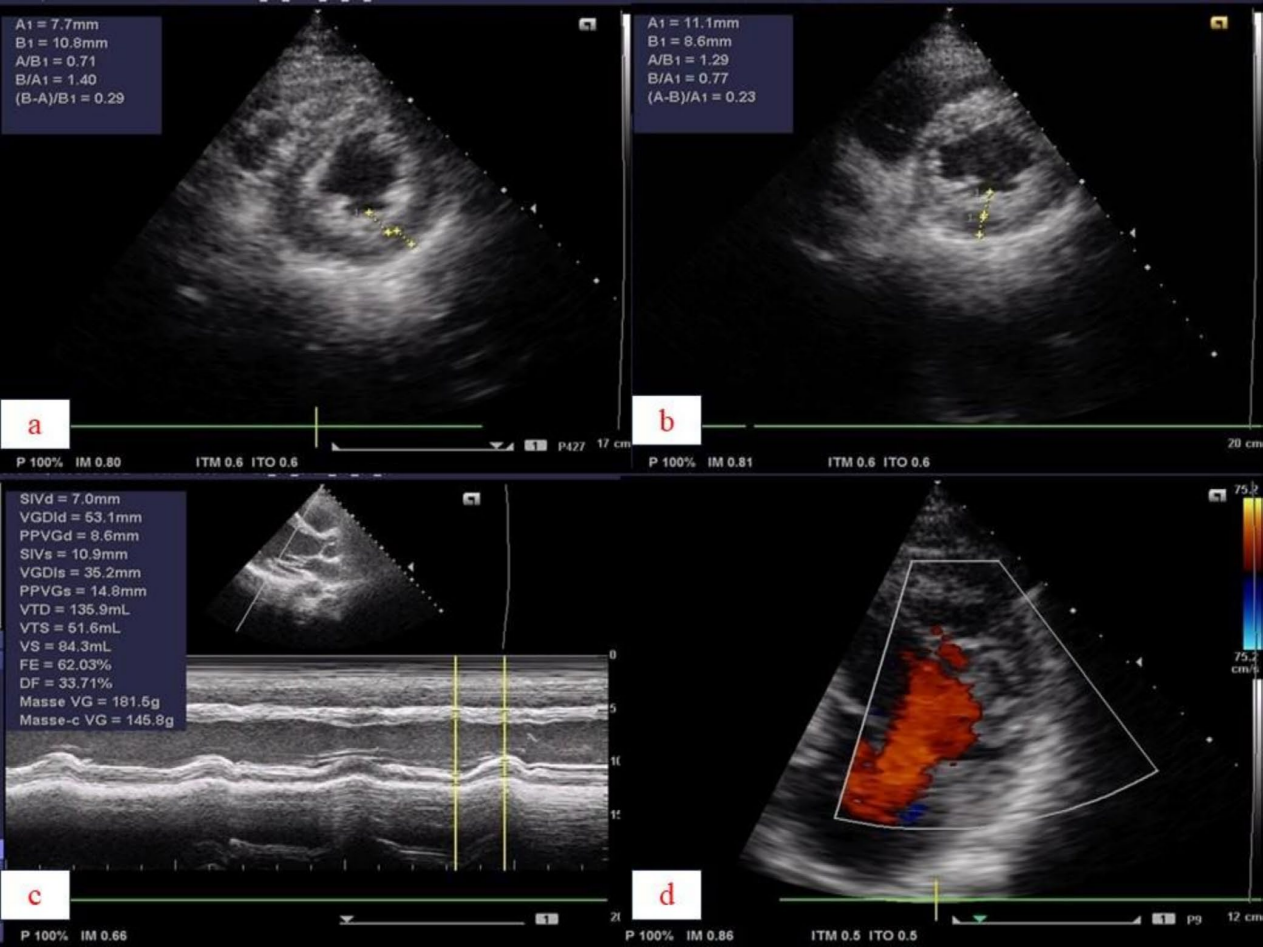
Echocardiography showing numerous trabeculations in the left ventricle. (a) Short‐axis asternal section showing a ratio of non‐compacted area to compacted area = 2 measured at the side wall. (b) Short‐axis asteroidal section showing a ratio of non‐compacted area to compacted area = 1.8 measured at the lower wall. (c) Long‐axis asternal section showing a FeVG calculated with Teicholz at 62%. (d) Color Doppler showing a vascularised recess inside the left ventricle.

**FIGURE 3:**
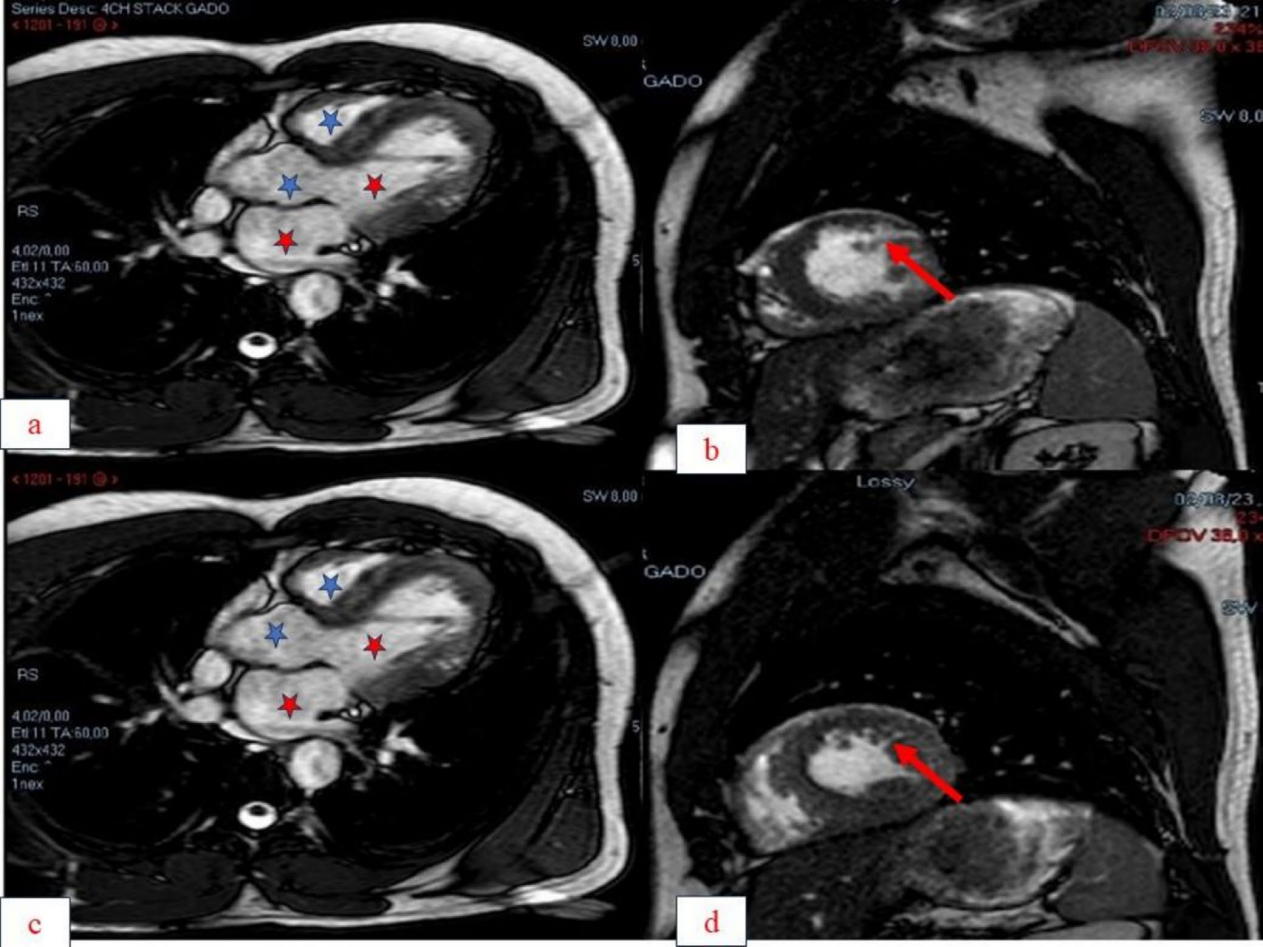
Cardiac MRI showing hypertrabeculation of the lateral, medial, and apical wall in favor of cardiomyopathy due to non‐compaction of the left ventricle. (a) and (c) Long‐axis incident view showing left cavities (red star) and right cavities (blue star). (b) and (d) Short‐axis view showing a vascularised recess (red arrow).

The emergency electrocardiogram revealed intolerable ventricular tachycardia (1er episode) at 220 cycles (Figure 1) per minute (cpm). The electrocardiogram after EEC and amiodarone showed a regular sinus rhythm at 81 cpm, a normal QRS axis at around +80 degrees, symmetrical negative T waves with lateral and inferior peaks, and a corrected QT of 410 ms. Echocardiography revealed numerous trabeculations in the left ventricle, with the ratio of non‐compacted area to compacted area = 2.4 in short‐axis section and 2.5 in apical 4‐cavity section. Good blood flow within the trabeculations was visualized by color Doppler, giving an overall echocardiographic appearance in favor of non‐compaction of the left ventricle (Figure 2). Magnetic resonance imaging (MRI) performed revealed hypertrabeculation of the medial and apical lateral walls. Non‐compacted myocardium measured 20.5 mm and compacted myocardium 6 mm. The ratio of non‐compacted to compacted myocardium was 3.04. LVEF was 64%. There was discrete hypokinesia of the lateral, medial, and apical wall. In conclusion, MRI showed hypertrabeculation of the lateral, medial, and apical wall in favor of cardiomyopathy due to non‐compaction of the LV (Figure 3). Laboratory tests were normal.

### 2.4 Diagnosis

The biological work‐up was normal, which allowed us to rule out acute myocarditis, especially as cardiac MRI did not show any subepicardial hypersignalling. Neither the Doppler echocardiogram nor the electrocardiogram showed any evidence of infarct scarring. The diagnosis was noncompaction of the left ventricle complicated by ventricular tachycardia in the presence of vascularised trabeculations with a ratio of non‐compacted area to compacted area greater than or equal to 2 on Doppler echocardiography and MRI.

### 2.5 Treatment

The patient received an external electric shock (EEC) at 200 J and was put on amiodarone.

### 2.6 Outcomes

The clinical and paraclinical evolution was favorable, with a return home after 1 week. He was readmitted after 4 months for a non‐tolerated ventricular tachycardia (2nd episode) following therapeutic non‐compliance. Regression of this last episode was obtained after a new CEE of 300 J and initiation of amiodarone.

## 3 Discussion

Left ventricular noncompaction cardiomyopathy is characterized by electrical abnormalities, including areas of low voltage and scarring, primarily related to the presence and extent of myocardial fibrosis rather than to noncompacted myocardium [5]. In this genetically diverse phenotype, the development of fibrosis contributes to an arrhythmogenic substrate underlying atrioventricular conduction diseases, supraventricular tachycardias, and ventricular tachycardias [6]. In addition, trabeculations reduce ventricular compliance, leading to diastolic dysfunction and hence coronary perfusion. The resulting ischemia is responsible for progressive fibrosis, with progressive degradation of systolic function and predisposition to DRT [7]. Within this spectrum, monomorphic ventricular tachycardia is the most frequently observed arrhythmia. It has been reported in 47% of symptomatic LVNC patients [2]. Atrial fibrillation is reported in 25% of patients with LVNC [8]. An electrophysiological study could be used to stratify risk and guide management in cases of VT. According to the recommendations for the treatment of heart failure with reduced EF (FE < 40%) of the European Society of Cardiology (ESC), the implantable defibrillator is indicated in the case of ischemic causes, and should only be used in the case of other etiologies to reduce the risk of sudden cardiac death [9]. It is not indicated for primary prevention of ventricular arrhythmias or sudden death in LVNC unless LVEF is severely reduced (< 35%) [10].

Although the incidence of ventricular arrhythmias and sudden cardiac death is high, there is little consensus on their management. To date, close monitoring using conventional cardiological diagnostic tools and symptomatic treatment, when necessary, constitute the therapeutic approach [11].

In addition to ventricular arrhythmias, the occurrence of coronary artery disease has been reported in many series. T. M., S. R., and K. M. have reported more than 07 cases of MI in patients with LVN [12]. Panduranga et al. reported a case of ACS without dyslipidemia and raised the hypothesis that the same gene responsible for NCVG could lead to a predisposition to coronary artery disease [13]. Martini et al. reported a series of cases of NCVG associated with familial dyslipidemia and coronary artery disease [14]. However, this explanation is not widely accepted, as the pathophysiology of the association between coronary artery disease and non‐compaction of the LV is still under investigation [15]. Coronary angiography would have shed light on a possible association with a coronary anomaly in our patient.

## 4 Conclusion

We report a case of non‐compaction of the LV associated with lateral ischemia complicated by severe TDR in the form of non‐tolerated VT. This rare and generally asymptomatic pathology may be revealed by systolic ventricular dysfunction, systematic embolic events, and ventricular arrhythmias or sudden cardiac arrest. The therapeutic decision depends on the patient's clinical picture and the physician's judgment. The implantable cardioverter defibrillator (ICD) appears to be an ideal tool, but its indications in this situation are limited to cardiac arrest, syncope, sustained VT, severe LVEF impairment, and a family history of sudden death.

## Author Contributions

**Martin Wendlassida Nacanabo:** conceptualization, funding acquisition, investigation, methodology. **André Arthur Taryètba Seghda:** supervision, validation, visualization. **Anna Tall/Thiam:** supervision, validation. **Georges Christian Millogo:** supervision, validation. **Valentin Nobila Yaméogo:** supervision, validation. **André Koudnoaga Samadoulougou:** supervision, validation, visualization. **Patrice Zabsonré:** supervision, validation.

## Funding

The authors have nothing to report.

## Consent

We have obtained the patient's consent for publication. Written informed consent was obtained from the patientto publish this report in accordance with the journal's patient consent policy.

## Data Availability

Service register.
